# Electroacupuncture Alleviates Chronic Pain-Induced Anxiety Disorders by Regulating the rACC-Thalamus Circuitry

**DOI:** 10.3389/fnins.2020.615395

**Published:** 2021-01-11

**Authors:** Zui Shen, Haiyan Zhang, Zemin Wu, Qiaoying He, Jinggen Liu, Yingling Xu, Shujing Yao, Xiaofen He, Yeqing Chen, Yi Liang, Boyi Liu, Yongliang Jiang, Junfan Fang, Junying Du, Xixiao Zhu, Mengwei Wu, Yuanyuan Wu, Jing Sun, Chi Xu, Jianqiao Fang, Xiaomei Shao

**Affiliations:** ^1^Key Laboratory of Acupuncture and Neurology of Zhejiang Province, Department of Neurobiology and Acupuncture Research, The Third Clinical Medical College, Zhejiang Chinese Medical University, Hangzhou, China; ^2^Qujiang District Hospital of Traditional Chinese Medicine, Quzhou, China

**Keywords:** chronic pain, pain-related anxiety, rostral anterior cingulate cortex, thalamus, circuitry, electroacupuncture

## Abstract

Anxiety is a common comorbidity associated with chronic pain, which results in chronic pain complexification and difficulty in treatment. Electroacupuncture (EA) is commonly used to treat chronic pain and anxiety. However, the underlying mechanisms of the EA effect are largely unknown. Here, we showed that a circuitry underlying chronic pain induces anxiety disorders, and EA can treat them by regulating such circuitry. Using chemogenetic methods, we found that chemogenetic activation of the rostral anterior cingulate cortex (rACC) glutamatergic output to the thalamus induced anxiety disorders in control rats. Then, chemogenetic inhibition of the rACC-thalamus circuitry reduced anxiety-like behavior produced by intraplantar injection of the complete Freund’s adjuvant (CFA). In this study, we examined the effects of EA on a rat model of CFA-mediated anxiety-like behaviors and the related mechanisms. We found that chemogenetic activation of the rACC-thalamus circuitry effectively blocked the effects of EA on chronic pain-induced anxiety-like behaviors in CFA rats. These results demonstrate an underlying rACC-thalamus glutamatergic circuitry that regulates CFA-mediated anxiety-like behaviors. This study also provides a potential mechanistic explanation for EA treatment of anxiety caused by chronic pain.

## Introduction

The International Association for the Study of Pain (IASP) defines pain as an unpleasant sensory and emotional experience associated with actual or potential tissue damage (Bushnell et al., 2013; Aydede, 2019; Meda et al., 2019). Clinically, approximately 20–50% of the patients with chronic pain have been reported to have anxiety (Vincent et al., 2015). Alternatively, there is accumulating evidence that anxiety may increase the sensation of pain or decrease pain tolerance (Becker et al., 2018; Gong et al., 2018). The mutual influence of pain and anxiety results in chronic pain aggravation and ineffective treatment. Mitigation of pain-related anxiety is thought to be a potential approach in the treatment of chronic pain. Electroacupuncture (EA), an improvement of traditional acupuncture, is commonly used to treat chronic pain (Vickers and Linde, 2014; Xiang and Wang, 2019) because of its safety, efficacy, and fewer side effects. Moreover, EA can also relieve anxiety (Pilkington, 2010). Our previous study showed that EA could mitigate anxiety-like behaviors induced by chronic pain in rats (Du et al., 2017). However, the mechanism by which EA regulates chronic pain-induced anxiety remains unclear.

Although substantial evidence shows that chronic pain leads to anxiety, the mechanisms underlying the chronic pain-induced anxiety are poorly understood. Research has revealed the roles of cortical neuronal networks in pain and anxiety. Among several cortical regions, the anterior cingulate cortex (ACC) has been reported to be involved in anxiety in both human and animal studies (Gross and Hen, 2004; Koga et al., 2015). Imaging studies have revealed increased ACC activity in patients with anxiety disorders (Osuch et al., 2000), and surgical lesions in the ACC or its chemical inactivation have produced anxiolytic effects in humans (Hornak et al., 2003) and animals. Despite strong evidence implicating the involvement of ACC in pain-related anxiety, little is known about how inputs from other brain regions engage ACC circuits.

The thalamus is the major source of nociceptive information transmission to the ACC (Sun et al., 2006; Yang et al., 2006). Lidocaine injections into or lesions of the medial thalamic nuclei can block the nociceptive responses of ACC neurons (Yang et al., 2006; Kiss et al., 2011). Imaging and electrophysiological studies in patients and animal models have also demonstrated that the thalamus, like the ACC, is hyperactivated during chronic pain (Rinaldi et al., 1991; Mao et al., 1993; Alshelh et al., 2016). Moreover, it has also been reported that activating thalamus outputs to the ACC exacerbates pain-related aversion (Meda et al., 2019). These studies raise the possibility that chronic pain might induce nagetive emotion through the thalamus-ACC circuit. But it is unknown whether ACC-thalamus circuitry was also involved in emotion regulation. Therefore, we aimed to investigate the role of the ACC-thalamus circuitry in chronic pain-induced anxiety and explore whether EA may have a therapeutic effect on chronic pain-induced anxiety disorders through regulation of this circuitry. We found that chemogenetic activation of the rostral ACC (rACC) inputs to the thalamus elicited anxiety-like behavior in control rats. However, chemogenetic inactivation of the rACC-thalamus circuitry reduced anxiety-like behavior produced by chronic pain induction via intraplantar injection of the complete Freund’s adjuvant (CFA). We also found that chemogenetic activation of the rACC-thalamus circuitry effectively blocked the therapeutic effect of EA on chronic pain-induced anxiety-like behavior in a CFA-induced chronic pain model in rats. These results reveal a novel circuit mechanism through which the rACC-thalamus circuitry influences chronic pain-induced anxiety. Moreover, our data also provide evidence that EA may interfere with chronic pain-induced anxiety disorder by regulating the rACC-thalamus circuitry.

## Materials and Methods

### Animals

Adult male Sprague-Dawley (SD) rats (7–8 weeks old, 250–300 g) were obtained from the Laboratory Animal Center of Zhejiang Chinese Medical University accredited by the Association for Assessment and Accreditation of Laboratory Animal Care (AAALAC). Three rats were housed together on soft bedding with food pellets and water *ad libitum*. Artificial 12 h light-dark cycles and proper temperature (lights on at 8:00 a.m.) were provided to allow for acclimation to the animal facility holdings for at least 5 days before any manipulation. All experiments were performed following the guidelines of the National Institutes of Health guidelines for the care and use of laboratory animals (NIH Publications No. 8,023, revised 1978) and approved by the Animal Ethics Committee of Zhejiang Chinese Medical University (ZSLL, 2017-183).

### Stereotaxic Injection

Adult male SD rats were anesthetized with isoflurane (4–5% induction, 1.5–2% maintenance). The head hair was then removed using a shaving machine, and the rats were fixed on a stereotaxic instrument (RWD, 68025, Shenzhen, China). The incisor bar was adjusted until the heights of lambda and bregma were equal to achieve a flat skull position. Erythromycin eye ointment was applied to the rat eyes to prevent corneal drying and pain from the use of strong light. A heating pad (RWD, 69000, Shenzhen, China) was used to maintain the body temperature at 37°C. According to Paxinos and Watson (2007), The Rat Brain in Stereotaxic Coordinates, bregma was defined as the point of intersection of the sagittal suture with the curve of best fit along the coronal suture, and lambda was defined as the midpoint of the curve of best fit along the lambdoid suture. The precise injection location of the rACC (AP, 2.76 mm; ML, 0.8 mm; DV, 1.4 mm) and the thalamus (AP, 1.80 mm; ML, 1.8 mm; DV, 5.1 mm) were obtained based on the distance between bregma and lambda. The skull craniotomy hole was made using a dental drill (WPI, OmniDrill35, Sarasota, FL United States), and injections were performed using a NanoFil 10 μL syringe via a micropipette connected to an Ultra Micro Pump (WPI, UMC4, Sarasota, FL United States) and its controller (WPI, UMC4, Sarasota, FL United States).

### Anterograde Tracer Virus Injection in the rACC

A total of 10 rats received an anterograde tracer virus (rAAV2/9-CaMKIIa-mCherry) microinjection in the right rACC, which can specifically label glutamatergic neurons. Rats were anesthetized with isoflurane (4–5% induction, 1.5–2% maintenance). Heart rate, temperature, and respiration were monitored throughout the surgery. The tracer was injected at 60 nL/min, and after each injection, the syringe remained *in situ* for 10 min and then removed. After a 6 weeks survival period, rats were deeply anesthetized and perfused with saline and 4% paraformaldehyde. Brains were removed, post-fixed overnight, dehydrated in increasing gradients of sucrose (15 and 30%), and preserved at −80°C. The distribution of the virus tracer was observed by fluorescence microscopy of coronal frozen sections, as described by Paxions and Watson.

### Chemogenetic Method

For virus-mediated tracing, rAAV2/9-CaMKIIα-mCherry was used (virus titer: 3.25 × 10^12^ vg/mL, 60 nL/min, 400 nL/injection; BrainVTA, China).

To specifically manipulate the glutamatergic neurons in the rACC output to the thalamus with mCherry, rAAV2/9-CaMKIIα-Dio-hM3D-mCherry (virus titer: 3.04 × 10^12^ vg/mL, 60 nL/min, 400 nL/injection) and rAAV2/9-CaMKIIα-Dio-hM4D-mCherry (virus titer: 3.38 × 10^12^ vg/mL, 60 nL/min, 400 nL/injection) were injected into the right rACC and rAAV2/R-CaMKIIα-Cre retrobeads (virus titer: 6.65 × 10^12^ vg/mL, 60 nL/min, 300 nL/injection) were injected into the thalamus.

The designer drug clozapine-N-oxide (CNO) (C0832, Sigma-Aldrich, St. Louis, MO) was administered 30 min before the behavioral assessment.

### CFA-Induced Chronic Pain-Related Anxiety Rat Model

**FIGURE 1:**
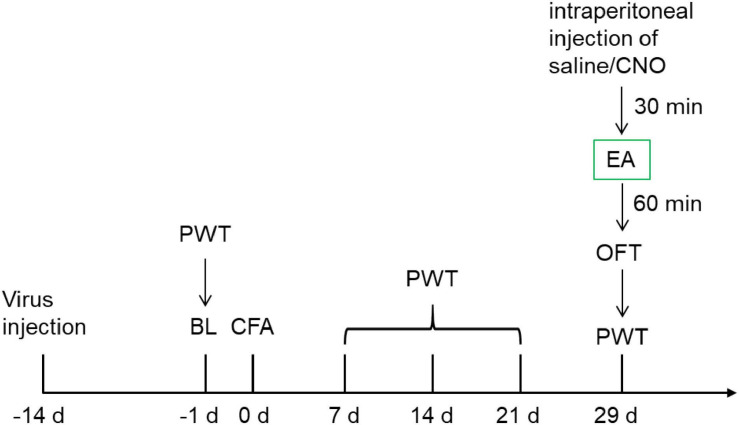
The schematic of the experimental procedure.

To establish the chronic inflammatory pain model in rats, 0.1 mL complete Freund’s adjuvant (CFA, Sigma-Aldrich) was suspended in an oil-saline (1:1) emulsion and injected subcutaneously into the plantar aspect of the left hind paw. The control rats were injected with 0.1 mL saline (0.9% NaCl). A schematic of the experimental procedure is shown in Figure 1.

### Open Field Testing

Twenty-nine days after CFA injection, the rats in all groups were subjected to open field testing to evaluate their spontaneous exploratory motor activity, and anxiety-like behavior.

The open field is a 1 × 1 m cube without a wooden box cover. The area was divided into 16 equal parts by the behavior tracking software acquisition and analysis system ANY-maze V6.14 (Stoelting, United States), which included 4 central and 12 peripheral areas. A camera was installed directly above the open field, and the image was adjusted to ensure a clear and smooth recording of the rats’ behaviors in the open field.

In the behavioral room, the temperature was 23–26°C, and the humidity was 40–60%. The open field was exposed to 20 lux illumination. The animals were placed in the testing room 30 min prior to the test to allow the rats to become acquainted with the new environment (acclimatization), thereby reducing stress. During the experiment, the rats were gently placed in the center of the open field. After the rats adapted to the experiment for 30 s, the ANY-maze was used to record and analyze the activity of the rats during the 5 min test. After the test was performed on each rat, the feces were collected and the test area was thoroughly cleaned with a cloth containing 10% alcohol. The test area was then dried with a cloth before performing the next experiment.

### Pain Withdrawal Thresholds

In the behavioral room, the temperature was 23–26°C, and the humidity was 40–60%. Before each test, the rats were placed in the room for 30 min for acclimatization, thereby reducing stress. Paw withdrawal thresholds (PWTs) were measured automatically with a dynamic plantar esthesiometer (model 37450; Ugo Basile, Comerio, Italy). A paw-flick response was elicited by applying an increasing vertical force (increased steadily from 0 to 50 g over 20 s) using a stainless steel probe (a straight 0.5 mm diameter shaft) placed underneath the mesh floor and focused at the middle of the plantar surface of the left hind paw. According to our previous study, PWTs were determined as the average of the last four measurements except for the first at intervals of 1 min (Shen et al., 2017). PWTs were recorded at baseline, 7, 14, 21, and 29 days after CFA injection. Moreover, all manipulations were performed by the same operator.

In the electroacupuncture experiment, a von Frey filament weighing 26 g was used to repetitively stimulate the middle of the plantar surface of the left hind paw for five times. The times that elicited a rapid withdrawal response were defined as the PWT.

### Electroacupuncture Treatment

The temperature of the room used for EA treatment was 23–26°C, and the humidity was 40–60%. The procedure of EA was carried out according to our previous study (Wu et al., 2017b). The bilateral Zusanli (ST36, 5 mm lateral to the anterior tubercle of the tibia) and Sanyinjiao (SP6, 10 mm proximal to the prominence of medial malleolus) acupoints were selected. Stainless steel acupuncture needles (0.18 mm in diameter, 13 mm in length) were inserted into the acupoints at a depth of 5 mm. The two ipsilateral needles were connected to the output terminals of a Hans Acupoint Nerve Stimulator (HANS-200E; Huawei Co., Ltd., Beijing, China). The EA parameters were set as follows: 100 Hz, square wave current output (pulse width, 0.6 ms) stimulation intensities ranging from 0.5 to 1.5 mA (increased by 0.5 mA every 20 min, for 60 min) delivered for 60 min. Animals were awake and calmed by placing their heads in black hoods with no physical restraint during EA treatment. In addition to the EA group, the other groups also adopted black hoods to reduce environmental differences. At 29 days after the CFA injection, EA was performed 30 min after CNO injection.

### Quantification of c-Fos Immunostaining

For the c-Fos assay, rats were sacrificed 90 min after CNO injection. Rats were deeply anesthetized and sequentially perfused with 0.9% (w/v) saline and 4% (w/v) paraformaldehyde. The rat brains were subsequently removed and post-fixed in 4% paraformaldehyde at 4°C overnight. After dehydration using 15% (w/v) and 30% (w/v) sucrose, coronal sections (30 μm) were cut on a frozen microtome (NX50; Thermo Fisher Scientific, MA, United States) and used for immunofluorescence. Sections were placed in a 37°C water bath, rewarmed for 1 h, washed with TBST six times, enclosed in 10% goat serum with 0.3% Triton X-100 for 1 h at 37°C, and then incubated with primary antibodies, including anti-c-Fos (1:800, rabbit, Abcam), at 4°C for 18 h. Subsequently, the sections were placed in a 37°C water bath, rewarmed for 1 h, washed with TBST six times, and finally incubated with secondary antibodies (Green, 111-545-144, Jackson) for 1 h at 23–26°C, and washed with TBST eight times. Then, the sections were incubated with 4′,6-diamidino-2-phenylindole (ab104139, Abcam), and for the nucleus staining, the sections were covered. Images were scanned using the Virtual Slide Microscope (VS120-S6-W; Olympus, Japan) for immunofluorescent staining. Images were analyzed using NIH Image J software (Bethesda, MD, United States). For the quantification of CaMKIIα + immunostaining, five inconsecutive brain slices (30 μm) containing the rACC were randomly selected.

### Statistical Analysis

All data are expressed as mean ± standard error (mean ± S.E.). Data from PWTs (at baseline, 7, 14, 21, and 29 days) were analyzed using repeated-measures analysis of variance (RM ANOVA). The remaining data were analyzed using one-way ANOVA. If the variance was uniform, Bonferroni test was used, and if not uniform, Dunnett’s T3 test was used. In all cases, results with *P* < 0.05 were considered statistically significant.

## Results

### The Thalamus Neurons Receive Glutamatergic Input From the rACC

**FIGURE 2:**
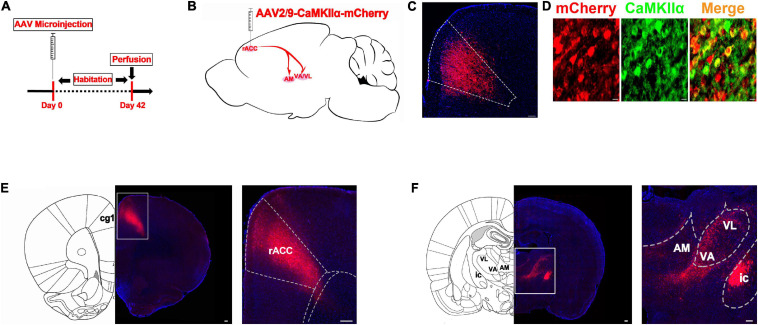
The thalamic neurons receive glutamatergic input from the rACC. **(A)** Scheme showing the configuration of the experiment. **(B)** Scheme for specifically labeled glutamatergic neurons in the rACC output to thalamus with mCherry. **(C)** The representative images of the whole rACC after virus injections. **(D)** mCherry signals were co-localized with the glutamatergic neuronal marker CaMKIIα in the rACC (scale bars: 20 μm). **(E)** Representative images of the virus injection site in the rACC after 6 weeks (scale bars: 1 mm in the middle of **D**, 200 μm in the right of **D**). **(F)** Representative images of the anteromedial thalamic nucleus (AM) and ventral anterior thalamic nucleus/ventrolateral thalamic nucleus (VA/VL) with rACC projections (scale bars, 1 mm in the middle of **E**, 500 μm in the right of **E**).

To identify the possible inferior brain regions connected to the rACC that could be involved in pain-induced anxiety, we first investigated the distribution of the mCherry signal after rACC infusion of rAAV2/9-CaMKIIα-mCherry in SD rats (Figures 2A,B). Six weeks after the virus injection, the mCherry signal in the rACC was co-localized with the glutamatergic neuron marker CaMKIIα (Figure 2C). We observed cell bodies with mCherry in the rACC (Figure 2D) and fibers with mCherry in the thalamus (Th), including the anteromedial thalamic nucleus (AM) and ventral anterior thalamic nucleus/ventrolateral thalamic nucleus (VA/VL). Since VA and VL were adjacent to each other, they were not subdivided in our study (Figure 2E).

### Chemogenetic Activation of the rACC Glutamatergic Output to the Thalamus Induces Anxiety-Like Behavior in Control Rats

**FIGURE 3:**
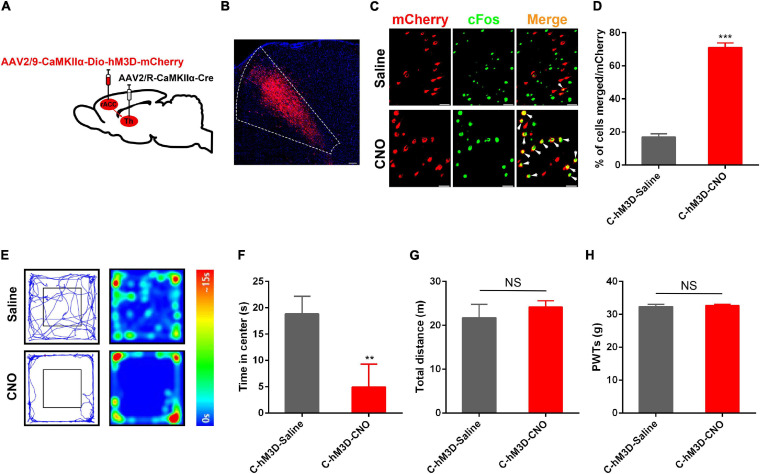
Anxiety disorders induced by specific activation of the rACC glutamatergic output to the thalamus in control rats. **(A)** Scheme showing configuration of the experiment. **(B)** The representative images of the whole rACC after virus injections. **(C)** Representative images of CaMKIIα-positive neurons (red) merged with c-Fos (green) in rats expressing the hM3D in the rACC (scale bars: 20 μm). **(D)** Percentage of total CaMKIIα co-expressing c-Fos in different groups (*n* = 4–5 rats per group). **(E)** Representative animal tracks from rats treated with saline/CNO at 43 days in the open field test. **(F)** Time in the center for rats treated with saline/CNO at 43 days in the OFT (*n* = 7 in the C-hM3D-Saline group; *n* = 11 in the C-hM3D-CNO group). **(G)** Total distance of rats treated with saline/CNO at 43 days in the OFT (*n* = 7 in the C-hM3D-Saline group; *n* = 11 in the C-hM3D-CNO group). **(H)** Paw withdrawal threshold in response to von Frey filaments (*n* = 11 in the C-hM3D-Saline group; *n* = 11 in the C-hM3D-CNO group). Data are presented as the mean ± SEM. **p < 0.01, ***p < 0.001 vs. C-hM3D-Saline group; NS, not significant.

The rACC and thalamus are activated in the state of pain and are strongly associated with negative emotions (Jensen et al., 2016; Zhu et al., 2020). Preliminary studies have shown theta-frequency phase-locking of single rACC neurons and synchronization with the medial thalamus (MT) in a rat model of pain (Wang et al., 2015). Meda et al. (2019) found that inhibition of the mediodorsal nucleus (MD) projecting ACC neurons produces aversion, specifically under chronic pain conditions. As described above, the rACC consisted of approximately 80% of pyramidal neurons. We found that thalamic neurons received direct rACC input, which prompted us to explore the functional contribution of the rACC-thalamus circuitry. We injected the monosynaptic retrograde transport virus rAAV2/R-CaMKIIα-Cre on the right side of the thalamus. Meanwhile, a Cre-dependent virus encoding the neuronal activator DREADD hM3D under the CaMKIIα promoter (rAAV2/9-CaMKIIα-DIO-hM3D-mCherry) was injected on the right side of the rACC (Figure 3A). Representative images of the rACC revealed the activation of glutamatergic neurons because of an increase in the co-expression of CaMKIIα and c-Fos after the intraperitoneal injection of CNO, compared with saline. We showed the percentage of total CaMKIIα cells co-expressing c-Fos in the Control(C)-hM3D-Saline and the Control(C)-hM3D-CNO groups (Figures 3C,D).

Forty-three days after stereotaxic injection, the control rat infected with hM3D-mCherry in the rACC-thalamus circuitry received an intraperitoneal injection of CNO (2 mg/kg), or the same volume of saline, 30 min before open field testing. Compared with saline-treated rats, we found that chemogenetic activation of the rACC-thalamus circuitry significantly increased the prevalence of anxiety disorders (Figures 3E–G). This is because these rats spent less time in the center area in the open field test. In addition, there was no difference in the PWTs between the two groups (Figure 3H).

### Chemogenetic Inhibition of Glutamatergic Neurons in the rACC-Thalamus Circuitry Reduced Anxiety-Like Behavior in a Rat Model of CFA-Induced Chronic Inflammatory Pain

**FIGURE 4:**
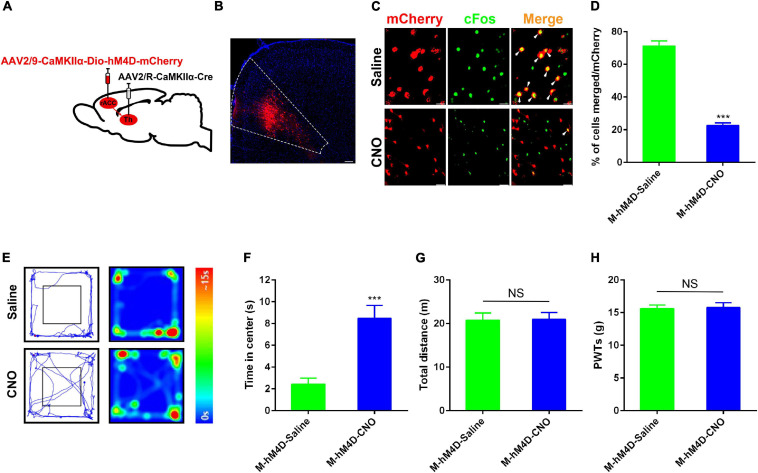
Specific inhibition of the rACC-thalamus circuitry alleviated anxiety disorders in the CFA rat. **(A)** Scheme of the virus injection in the rACC projecting to the thalamic neurons with rAAV2/9-CaMKIIα-DIO-hM4D-mCherry and rAAV2/R-CaMKIIα-Cre. **(B)** The representative images of the whole rACC after virus injections. **(C)** Representative images of CaMKIIα-positive neurons (red) merged with c-Fos (green) in rats expressing the hM4D in the rACC (scale bars: 20 μm). **(D)** Percentage of total CaMKIIα coexpressing c-Fos in different groups (*n* = 5 rats per group). **(E)** Representative animal tracks from rats treated with CNO at 43 days in the OFT. **(F)** Time spent in the center of the field by the rats treated with saline/CNO at 43 days in the OFT (*n* = 10 in the M-hM4D-Saline group, *n* = 12 in the M-hM4D-CNO group). **(G)** Total distance covered by the rats treated with saline/CNO 43 days in the OFT (*n* = 10 in the M-hM4D-Saline group, *n* = 12 in the M-hM4D-CNO group). **(H)** Paw withdrawal threshold in response to von Frey filaments (*n* = 15 in the M-hM4D-Saline group, *n* = 14 in the M-hM4D-CNO group). Data are presented as the mean ± SEM. ***p < 0.001 vs. M-hM4D-Saline group; NS, not significant.

Since activation of the rACC-thalamus circuitry in control rats induced anxiety, we investigated whether the inhibition of the excitability of the rACC-projecting thalamic neurons suppressed pain-induced anxiety disorders. A Cre-dependent virus encoding the neuronal activator DREADD hM4D under the CaMKIIα promoter (rAAV2/9-CaMKIIα-DIO- hM4D-mCherry) virus was injected on the right side of the rACC, and rAAV2/R-CaMKIIα-Cre was injected in the right side of the thalamus (Figure 4A). Representative images of the rACC showed the inhibition of glutamatergic neurons, because of the decreased co-expression of CaMKIIα and c-Fos after the intraperitoneal injection of CNO, compared with saline. We showed the percentage of total CaMKIIα-positive cells co-expressing c-Fos in the Model(M)-hM4D-Saline and the Model(M)-hM4D-CNO groups (Figures 4B,C). CFA was injected into the left footpad of rats 2 weeks after virus injection. In the same way, at day 43 after stereotaxic injection, the CFA rat with hM4D-mCherry in the rACC-thalamus circuitry received intraperitoneal injection of CNO (10 mg/kg) or saline 30 min before open field testing. Subsequently, the rats were tested for PWTs. Compared with saline-treated rats, we found that chemogenetic inhibition of rACC projecting to thalamic neurons significantly decreased anxiety-like behavior in CFA rats (Figures 4E–G). Interestingly, rats spent an increased time in the open center area, while the PWTs remained unchanged (Figure 4H). Figures 3, 4 show that anxiety-like behavior is related to the rACC-thalamus circuitry, and inhibition of the rACC-thalamus circuitry could relieve CFA-induced anxiety-like behavior.

### EA Effectively Reduces CFA-Induced Anxiety-Like Behavior in Rats

**FIGURE 5:**
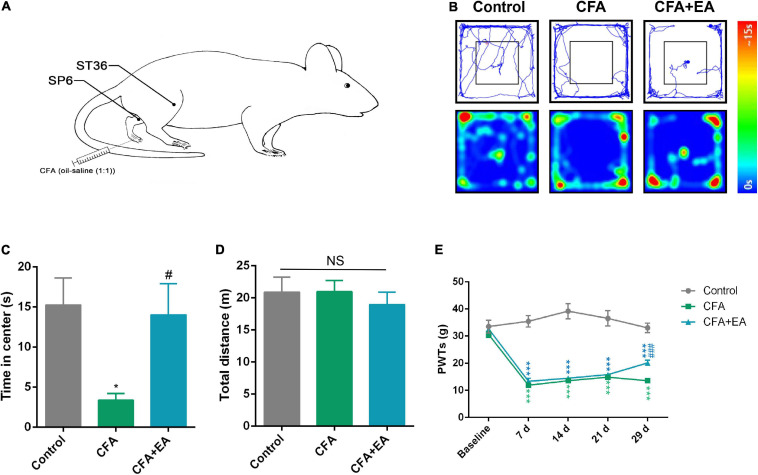
EA effectively reduces pain-related anxiety behavior in a rat model of CFA-induced chronic inflammatory pain. **(A)** Schematic of the EA treatment. **(B)** Representative animal tracks in control, CFA, and CFA + EA groups for 29 days in the OFT. **(C)** Time spent in the center of the field by the rats in the control, CFA, and CFA + EA groups for 29 days in the OFT (*n* = 7 in the control group; *n* = 8 in the CFA group; *n* = 7 in the CFA + EA group). **(D)** Total distance covered by the rats in control, CFA, and CFA + EA groups for 29 days in the OFT (*n* = 7 in the control group; *n* = 8 in the CFA group; *n* = 7 in the CFA + EA group). **(E)** Paw withdrawal threshold in response to von Frey filaments (*n* = 10 in the control group; *n* = 10 in the CFA group; *n* = 8 in the CFA + EA group). Data are presented as the mean ± SEM, *p < 0.05, ***p < 0.001 vs. control group; *^#^p* < 0.05, *^###^p* < 0.05 vs. CFA group; NS, not significant.

We established a rat model of CFA-induced chronic inflammatory pain by injecting 0.1mL oil-saline (1:1) emulsion into the plantar aspect of the left hind paw (Figure 5A). The pain withdrawal threshold decreased significantly in CFA-treated rats compared with saline-treated rats from day 7 onward and lasted a minimum of 29 days. These results were consistent with previous reports, indicating the successful establishment of CFA-induced chronic inflammatory pain in rats. After 29 days of CFA injection, the rats were assessed using open field testing. The model rats spent less time in the center area than the control group, which is indicative of chronic pain-induced anxiety-like behavior (Figures 5B–D). The EA group was treated with 100 Hz EA for 1 h on bilateral ST36 and SP6 acupoints located on the hind limbs of the rats. Treatment with EA improved anxiety-like behavior in CFA-treated rats, as they spent more time in the center of the open field (Figures 5B–D). Meanwhile, EA produced an analgesic effect compared with CFA-treated rats (Figure 5E). Figures 4, 5 demonstrate that EA and inhibition of the rACC-thalamus circuitry have similar effects on relieving CFA-induced anxiety-like behavior.

### The Effect of EA on CFA-Induced Anxiety-Like Behavior Is Attenuated by Chemogenetic Activation of the rACC-Thalamus Circuitry

**FIGURE 6:**
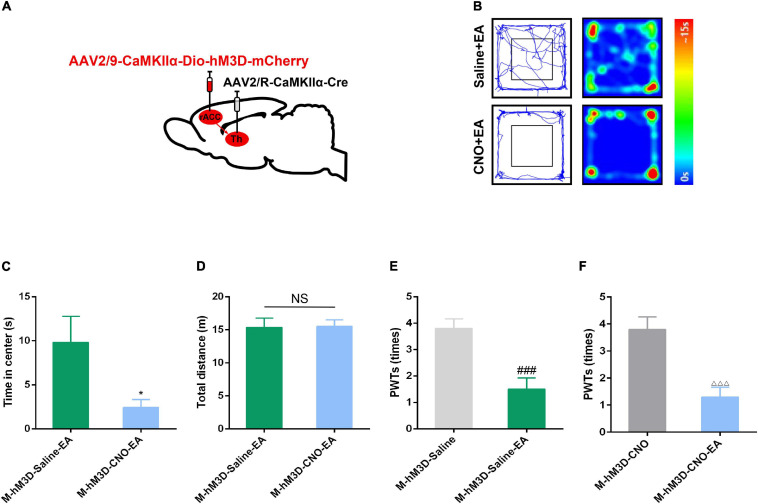
The effect of EA on pain-related anxiety disorders could be reversed by specific activation of the rACC-thalamus circuitry. **(A)** Scheme for specific injection of rACC-projecting thalamus neurons with rAAV2/9-CaMKIIα-DIO-hM3D-mCherry. **(B)** Representative animal tracks of rats treated with saline/CNO at 43 days in the OFT. **(C)** Time spent in the center of the field by the rats treated with saline/CNO at 43 days in the OFT (*n* = 15 in the M-hM3D-Saline-EA group; *n* = 14 in the M-hM4D-CNO-EA group). **(D)** Total distance covered by the rats treated with saline/CNO 43 days in the OFT (*n* = 15 in the M-hM4D-Saline-EA group; *n* = 14 in the M-hM4D-CNO-EA group). **(E)** Paw withdrawal threshold in response to von Frey filaments (*n* = 10 in the M-hM3D-Saline-EA group; *n* = 10 in the M-hM4D-CNO-EA group). **(F)** Paw withdrawal threshold in response to von Frey filaments (*n* = 10 in the M-hM3D-Saline-EA group; *n* = 10 in the M-hM4D-CNO-EA group). Data are presented as the mean ± SEM. *p < 0.05 vs. M-hM3D-Saline-EA group; *^###^p* < 0.001 vs. M-hM3D-Saline group; ^△^^△^^△^ *p* < 0.001 vs. M-hM3D-CNO group; NS, not significant.

To verify whether EA could inhibit CFA-induced anxiety-like behavior by inhibiting rACC-thalamus circuitry. The Cre-dependent virus encoding the neuronal activator DREADD hM3D under the CaMKIIα promoter (rAAV2/9-CaMKIIα-DIO- hM4D-mCherry) virus was injected to the right side of the ACC and the rAAV2/R-CaMKIIα-Cre was injected to the right side of the thalamus (Figure 6A). CFA was injected subcutaneously into the plantar aspect of the left hind paw after 2 weeks.

Interestingly, we found that chemogenetic activation of glutamatergic neuronal activity in the rACC-thalamus circuitry reversed the inhibitory effect of EA on chronic pain-induced anxiety (Figures 6B–D). However, compared with the M-hM3D-Saline and the M-hM3D-CNO groups, EA could relieve CFA-induced tactile allodynia in the M-hM3D-Saline-EA and in the M-hM3D-CNO-EA groups (Figures 6E,F). These data showed that EA may relieve CFA-induced anxiety-like behavior by inhibiting the rACC-thalamus glutamatergic circuitry, but this circuitry may not be involved in the effect of EA on CFA-induced tactile allodynia.

## Discussion

In this study, we showed that chemogenetic control of the activation of the rACC-projecting thalamic glutamatergic neurons significantly modulates chronic pain-related anxiety disorders bilaterally, with no change in pain perception. EA can alleviate chronic pain and pain-related anxiety disorders, and the effect of EA on pain-related anxiety disorders can be reversed by activation of the rACC-projecting thalamic glutamatergic neurons, with no change in pain threshold. These results demonstrate that there is a way to separate pain perception and pain-related anxiety disorders by selecting the rACC glutamatergic projecting thalamic neurons. EA may interfere with chronic pain-induced anxiety disorders by regulating the rACC-thalamus circuitry.

Anxiety is a common comorbidity associated with chronic pain, which results in chronic pain aggravation and increased difficulty of treatment. It has been reported that several circuitries formed by different brain regions are required for the induction of anxiety disorders (McLaughlin et al., 2017; Robinson and Pike, 2019). However, the exact circuitry underlying anxiety disorders induced by chronic pain has not yet been defined. The advantage of studying the neural circuitry mechanism of anxiety disorders induced by chronic pain is that it can reveal convergent pathological behavioral consequences. In this study, we focused on the rACC and thalamus, which are critical in anxiety disorders and other negative emotions (Collins et al., 2018; Berry et al., 2019).

The rACC is a higher center for processing affective emotions (Yao et al., 2019). In the central nervous system, the rACC integrates pain and emotional information from the frontal cortex, thalamus, and amygdala (Zheng et al., 2018; Liu et al., 2019). It is mainly involved in the regulation of pain emotion and has a limited effect on pain sensation. Neuroanatomical evidence in previous studies has not described the relationship between the rACC and its descending afferent nuclei. Activation of glutamatergic neurons in the rACC will induce an aversive teaching signaling (Johansen and Fields, 2004). A previous study found that excitotoxin-induced rACC lesion reduced acute formalin-induced conditional location aversion (F-CPA) without altering acute formalin-induced pain sensation (Johansen et al., 2001). The rACC-injected NMDA receptor antagonist significantly inhibited F-CPA in rats; however, it did not reduce acute formalin-induced nociceptive behaviors (Ren et al., 2006). The engagement of the rACC circuitry has been shown to be associated with the modulation of the affective component without altering pain intensity (Singh et al., 2020). Interestingly, our present study also demonstrated that chemogenetic inhibition of glutamatergic neurons in the rACC-thalamus circuitry only reduced anxiety disorders without changing the rats’ pain thresholds induced by CFA. Consistent with previous results, these results demonstrated that there was a separation between the affective and sensory components of pain (Sellmeijer et al., 2018). The rACC mainly deals with pain-related anxiety behavior rather than pain perception.

In addition, there is scarce evidence on the circuits involved in the modulation of pain-related anxiety disorders. We first used an anterograde tracing virus to identify the input region of glutamatergic projecting nuclei from the rACC and elucidated that the thalamus, including the anteromedial thalamic nucleus (AM) and the ventral anterior thalamic nucleus/ventrolateral thalamic nucleus (VA/VL), was the subordinate projecting nucleus. Among the subordinate nuclei receiving projections from the rACC, the nucleus of the VA/VL of the thalamus is related to pain cognition and decision (Hooks et al., 2013). Previous studies have found a positive correlation between high thalamic activity and anxiety (Geng et al., 2018). The level of anxiety increased in rats with electrolytic lesions in the thalamic reticular nucleus (El Boukhari et al., 2019). Adult rats with bilateral medial dorsal (MD) lesions in the early postpartum period showed impairment of recognition memory and increased anxiety-like behavior (Ouhaz et al., 2017). Excessive activation of the VA and MD nuclei in the thalamus leads to obsessive-compulsive disorder (OCD), which is often associated with anxiety in patients with OCD (Rotge et al., 2012). Using fMRI, researchers have found a positive correlation between trait anxiety and activation in the medial thalamus during pain encoding (Tseng et al., 2017). During alcohol consumption, anxiety symptoms were found to be positively correlated with thalamus activation (Feldstein Ewing et al., 2010). The paraventricular nucleus of the thalamus (PVT) is involved in regulating emotional behaviors, including anxiety and fear (Li et al., 2010). Thus, it is necessary to characterize the functional connections in the rACC-thalamus circuitry.

In the present study, we demonstrated that the rACC-thalamus glutamatergic circuitry mediates pain-induced anxiety disorders. We used chemical genetic methods to activate the rACC glutamatergic neuron input to the thalamus. In this part of the experiment, CFA was injected into the left hind foot of rats to induce chronic inflammatory pain. Many studies have shown that CFA plantar injection can cause redness, swelling, fever, and hypersensitivity of the plantar, and that the pain lasts for at least 1 month. The chronic pain model showed anxiety-like behavior with the extension of time, which further supported the existence of emotional disorders caused by pain.

Based on our results, we found that activation of the glutamatergic neurons in the rACC-thalamus circuitry increased anxiety disorders in control rats, and inhibition of the rACC-thalamus circuitry decreased anxiety disorders in CFA rats, indicating a sustained effect of rACC-thalamus circuitry on pain-related anxiety. Glutamate is the most abundant excitatory neurotransmitter in the central and peripheral nervous systems of mammals (Ribeiro et al., 2017) as well as the dominant neurotransmitter in the excitatory transmission process in the central nervous system (Reiner and Levitz, 2018). In our results, glutamatergic neurons could participate in the induction and maintenance of pain-related anxiety, which indicates a functional link between the rACC and thalamus. The rACC and thalamus are both the hubs of reception and transmission of anxiety in the brain. Although our results indicate that the rACC-thalamus circuitry is associated with chronic pain-related anxiety disorders, it is reasonable to suppose that the change in anxiety disorders controlled by the rACC-thalamus circuitry will affect other behaviors, including motivation and motion. It has been reported that most VL neuronal discharge can promote motor cortex-related activities (Marlinski et al., 2012). Indeed, motor function was markedly improved, with mood stabilization, in patients with Tourette syndrome (TS) that underwent deep brain stimulation (Huys et al., 2016). These results support the hypothesis that the rACC-thalamus circuitry is a specific target for bidirectional regulation in pain-induced anxiety. However, it is necessary to explore the downstream nuclei in future studies.

Clinically, chronic pain is a serious public health problem, which can have a negative impact on the quality of life and is associated with substantial socio-economic costs (Blyth et al., 2015). In general, chronic pain is accompanied by negative emotions such as anxiety, depression, aversion, and avoidance (Gerrits et al., 2014). Therefore, analgesics are used in combination with anti-anxiety drugs, tranquilizers, or antidepressants to alleviate affective pain (Torrance et al., 2018). EA is widely used in the treatment of pain, and it is often used to treat chronic pain-induced negative emotions in the clinical setting (Pei et al., 2019; Zhong et al., 2019).

According to traditional Chinese medicine, SP 6 can promote qi circulation and alleviate negative emotion. In our previous study, we observed that EA on ST 36 + SP 6 acupoints could inhibit CFA-induced pain aversion (Wu et al., 2017a). Moreover, by comparing the two acupoint combinations (ST36 + SP6 vs. LI11 + TE5), we found that EA on ST36 + SP6 could attenuate depression-like behavior (Wu and Jiang, 2015). In our study, subjecting the rats to 100 Hz of EA for 1 h and reduced their pain thresholds and anxiety disorders. When the rACC glutamatergic neuron output to the thalamus was activated, the effect of EA on pain-related anxiety disorders was reversed, but no changes in pain perception were observed. This suggests that EA may regulate pain-related anxiety disorders by modulating the activation of the rACC-thalamus circuitry. The circuitry of the rACC-thalamus may be a novel target for the treatment of pain-related anxiety in the clinical setting. It is possible that other neurocircuitries in the brain control multiple responses because of the effect of EA, as observed in our study.

In conclusion, we demonstrated a novel circuit mechanism through which the rACC-thalamus circuitry influences chronic pain-induced anxiety. Furthermore, this study provides evidence that EA may interfere with chronic pain-induced anxiety disorder by regulating the rACC-thalamus circuitry.

## Data Availability Statement

The original contributions presented in the study are included in the article/supplementary material, further inquiries can be directed to the corresponding author/s.

## Ethics Statement

The animal study was reviewed and approved by the Animal Ethics Committee of Zhejiang Chinese Medical University (ZSLL, 2017-183).

## Author Contributions

ZS and HZ performed data analysis and applied the chemogenetic method. ZW and QH performed surgeries. XS and JiF designed the experimental protocols. JL, CX, JuF, and JD performed manuscript writing. YX and SY performed paw withdraw threshold testing. XH and YC performed the open field test. BL and YL performed the immunostaining. XZ and MW performed electroacupuncture treatment. YW, JS, and YJ performed statistical analysis. All authors contributed to the article and approved the submitted version.

## Conflict of Interest

The authors declare that the research was conducted in the absence of any commercial or financial relationships that could be construed as a potential conflict of interest.
